# Vaccination Papers

**Published:** 1872-04

**Authors:** 


VACCINATION PAPERS.—In the *British Medical Journal*, Dec. 23, 1871, Dr. T. J. Preston gives a valuable suggestion respecting a new method of lymph preservation. It is certainly portable, very convenient, and the doctor says certain in its effects. He paints a common sheet of note-paper with the lymph, fresh from the vesicle. The lymph soon dries, and the paper is ready for use. When required, a minute piece may be torn off the sheet, and, after being breathed upon slightly, should be stuck upon the freshened surface. If the paper be required to be kept for any length of time, it should, after being charged, be covered with a thin coating of white of egg.—*Detroit Review of Medicine and Pharmacy; Nashville Medical Journal.*

